# Assessment of the ability DS-EIA-HIV-AB-TERM assay correctly identify long-standing and recent HIV infection

**DOI:** 10.1186/1471-2334-14-S2-P70

**Published:** 2014-05-23

**Authors:** YE Zagryadskaya, VF Puzyrev, AN Burkov, DA Neshumaev, TI Ulanova, Irina Sharipova

**Affiliations:** 1 RPC “Diagnostic Systems”, Nizhny Novgorod, Russia; 2 DSI S.r.l. Saronno (VA), Italy; 3 Krasnoyarsk Regional Center of AIDS Prevention, Krasnoyarsk, Russia

## Background

The assessment of newly acquired human immunodeficiency virus type 1 (HIV-1) infection, or incidence, provides important information for public health programs and is crucial for understanding the status of the epidemic and providing information regarding the impact of prevention measures. The aim of this study was to evaluate the ability of the assay DS-EIA-HIV-AB-TERM correctly identify long-standing and recent HIV infection in samples with the preliminary defined time of HIV-1 infection.

## Materials and methods

The assay DS-EIA-HIV-AB-TERM intended for the identification of recently acquired HIV-1 infection was used in the study. Efficiency assessment of the test was carried out using serum samples (n=239) with the preliminary defined time of HIV-1 infection which was identified based on HIV algorithm testing: recent infection samples (n=150), long-standing infection (n=89, including samples with a low CD4+ T cell count) ("Krasnoyarsk Regional center of AIDS prevention", "Nizhny Novgorod Regional center of AIDS prevention", Russia). Additionally the assay assessment was carried out using seroconversion panels (n=32) (SeraCare, ZeptoMetrix, USA).

## Results

The assay is able to define as recent infection 100% of samples with seroconversion profile. Among samples with the preliminary defined time of HIV infection the accuracy of testing was: 93.3% - for recent (up to 9 months), 97.8% - for long-standing infection (9 month and more). All serum samples with late-stage infection (low CD4+ T cell count) were determined correctly.

## Conclusion

Our data demonstrated high efficiency of the kit DS-EIA-HIV-AB-TERM to identify both recently acquired and long-standing infection among samples from HIV type 1 seropositive persons.

